# Implementation of New Technologies in an Aged Care Social Day Program: Mixed Methods Evaluation

**DOI:** 10.2196/60297

**Published:** 2025-05-12

**Authors:** Dannielle Post, Kathleen Whitson, Gaynor Parfitt

**Affiliations:** 1 Alliance for Research in Exercise, Nutrition, and Activity, Allied Health and Human Performance, University of South Australia, City East Campus, Frome Road, GPO Box 2471, Adelaide, 5001, Australia, 61 8 830 21212

**Keywords:** aged care, older adults, interactive robots, social engagement, evaluation, geriatric, robot, day program, perception

## Abstract

**Background:**

Australia’s aging population is looking to age in place, accessing care alternatives external to the traditional model of residential aged care facilities. This evaluation is situated in a Social Day Program, delivered by an aged care organization. It is designed to cater for people living with dementia, located in an environment equipped with new technologies including age-specific interactive computer gaming, social robots, sensory stimulation, and virtual reality. The technologies are designed to support older adults, enabling them to stay connected and maintain physical and cognitive functioning, independence, and quality of life.

**Objective:**

This project aimed to undertake a multifaceted evaluation of the implementation of the new technologies, including an exploration of the barriers and enablers to uptake. The key issue is how to enhance the potential for optimizing the use of these technologies in the Social Day Program environment, to help inform decision-making regarding the implementation of these technologies at the organization’s other sites, and future investment in such technologies by aged care organizations generally.

**Methods:**

Observation of technology use within the organization was conducted over a 16-week period. Surveys and semistructured interviews were used to collect information from staff related to their experiences with the technology. Thematic analysis was used to analyze the interviews. Data were triangulated across the sample.

**Results:**

Forty-eight observation periods were completed, totaling 126.5 observation hours. Technology use by clients was observed on 24 occasions, for 22 (17.4% of the observation time) hours. Nineteen staff completed surveys. Nearly three-quarters (n=14) of the staff perceived there to be barriers to the clients’ use of technology, and 18 (95%) staff reported that they assisted clients to use the technology. Ten (53%) staff reported receiving training to use the technology and feeling confident in their knowledge of the technology to assist clients in using it. Twelve staff members participated in an interview. Key themes identified from the interview data were: technology has potential but is not for everyone, incorporating the subtheme technology as a placation tool, staff knowledge and confidence, and technology functionality and support.

**Conclusions:**

This evaluation identified that technology was not being used for the purposes of enrichment or experience enhancement, nor extensively. Multiple barriers to the implementation and sustained use of the technology items were identified. Recommendations to improve implementation and promote sustained use of technology, based on the findings of this evaluation and evidence from the literature, may apply to other organizations seeking to implement these technologies in similar programs.

## Introduction

### Background

With a shift to consumer-directed care, and Australia’s aging population looking to age in place and access a range of supported care alternatives outside the traditional model of residential aged care facilities, there are a lot of new offerings for consumers. Often funded through the Australian government–supported Aged Care Community Packages, these offerings can include in-home care and home support services or colocated aging-specific services in centralized hubs. Services include Dietetics, Allied Health Group Programs, Exercise Physiology, Gym and Physical Activity, Massage, Music Therapy, Occupational Therapy, Physiotherapy, Podiatry, Speech Pathology, Gerontology, and Social Day Programs.

ECH Inc (hereinafter referred to as the organization) is a South Australian retirement living and in-home care services provider established in 1964 and based in the state of South Australia. The “profit for purpose” organization’s mission is “helping people to live confidently and independently, and to get more out of life” [1]. This project was situated in one of the organization’s Social Day Programs, which occur at 4 centers across metropolitan South Australia. The Social Day Program involved in this evaluation is situated within a proof-of-concept Care Hotel that was purpose-designed to provide care givers respite options with 8 short-stay suites for people living with dementia or who may be recovering from surgery. These Social Day Programs, provided on weekdays between 9 AM and 3 PM, are designed to cater for community-dwelling people living with dementia or memory loss and are located in environments where the clients have space to engage socially with others or have supported, quiet alone time. Clients can be dropped off at and picked up from the Social Day Program by family members or transported to and from the Social Day Program by a bus provided by the organization. Program staff are trained to ensure that each client’s interests and abilities are met, with the provision of activities that are enjoyable and meaningful. Participation in the Social Day Program is also available to Care Hotel guests, as is access to the space outside the Social Day Program hours (evenings and weekends). This space is equipped with new technologies that are designed to support older adults as they age, enabling them to stay connected and maintain physical and cognitive functioning, independence, and quality of life [2-5]. These technologies are varied and includes an age-specific interactive computer gaming console, “Obie” that projects interactive games onto a surface (table or floor) to encourage active play; a relaxation chair in a sensory stimulation room; virtual reality (VR) headsets (the Odysee system, with virtual experiences including travel, museums, and hot air ballooning); white noise bubble tubes (tubes with color changing lights, filled with bubbles, that are intended to provide visual distraction and calm); 2 robotic dolls; and a robotic sensory cat.

Systematic reviews support the effectiveness of these technologies, with interactive computer gaming improving physical and psychological functioning [6]; interactions with social robots positively affecting agitation, anxiety and loneliness, medication consumption, and quality of life [5]; and VR technology improving physical health outcomes [7] and cognition, memory, and depression [8] for older adults. The implementation of such technologies in the aged care environment is not without its challenges. Other studies have identified organizational factors, such as funding and staff engagement or knowledge, client-specific issues such as frailty, dementia, or limited prior exposure to technology, inappropriate or unsuitable technologies, or resistance from family members [9]. Similar barriers were identified in a systematic review of VR technology, with the addition of the potential for cybersickness during use also perceived to be a barrier [10]. Research focused on social and assistive robots has identified concerns around the potential for infantilization or loss of dignity for older adults who use them [11]. The items chosen by the organization were selected due to the evidence supporting their effectiveness and with the intention of supporting engagement and enrichment for their clients. Providing a variety of technologies, rather than a single type of technology, was intended to broaden the appeal of technology to clients.

### This Study

The organization requested an extensive review of the use of these technologies in this unique setting. This research aimed to undertake a multifaceted evaluation of the implementation of technologies at the Social Day Program, including an exploration of the barriers and enablers to the use of technology within the Social Day Program. The key issue, and the focus of this paper, is identifying how to enhance the potential for optimizing the use of these technologies at the organization, making recommendations to help inform decision-making regarding the implementation of these technologies at other sites, and future investment in new technologies for the organization’s clients.

The evaluation was intended to answer the following research questions:

1. What is the utility of these technologies?
2. What are the barriers and enablers to the uptake of these technologies?
3. What is the impact (physical, psychosocial, and quality of life) of technology use experienced by the clients, as perceived by the staff?

## Methods

### Study Design

Observation of technology use within the Social Day Program was conducted over 16 weeks by a research assistant (RA), with periodic support from undergraduate physiotherapy students completing a health promotion placement with the evaluation team. Surveys and semistructured interviews were used to collect information from staff related to their experiences with the technology. Data were triangulated across the sample, with observation, survey, and interview data integrated and compared to establish alignment and contrast among the findings of each method.

### Participants

Participants were staff working in the organization’s Care Hotel or Social Day Program. Staff provided written informed consent to participate in the evaluation. For the observation component, participants included clients of the Social Day Program and clients of the Care Hotel who were accessing the Social Day Care space.

### Observation of Technology Use

Observation of the use of technology by staff, Social Day Program clients, and Care Hotel guests using the Social Day Program space was undertaken between July and November 2022. Observation periods of 4 hours each, either morning and overlapping early afternoon or late morning until the end of the day, occurred during the Social Day Program sessions across the week, but also after hours and on weekends to ensure capture of use of technology outside of typical program activity periods. An observation checklist was used to capture data related to technology-user type (ie, client, family member or carer, staff, or other), technology item, duration of use, concurrent use of an item by multiple participants, support or assistance provided, emotional impacts, communication, physical impacts, and other notable information. This checklist was developed by the evaluation project team in consultation with the Stakeholder Advisory Group and piloted by the RA and placement student observers, with checklist items modified as required throughout piloting.

### Surveys

Consenting staff completed an anonymous electronic survey (Qualtrics). Survey items were initially drafted by the evaluation team and refined using a co-design approach in collaboration with the Stakeholder Advisory Group. The Stakeholder Advisory Group was comprised of members of the organization and evaluation project team and a representative from each of the 3 stakeholder-participant groups (clients, family members or carers, and staff of the Social Day Program) and was formed to ensure consumer awareness and the suitability and feasibility of evaluation methods. The staff survey was intended to identify the perceived implications for staff involved in implementing the technology and supporting clients to use the technology.

Survey questions collected information about participant demographics, staff assistance with technology items, length of time staff assisted clients with the technology, and most- and least-assisted technology items. Perception of impact were collected across 6 domains relative to each of the items of technology: social engagement, cognitive awareness, communication, mood state, activities of daily living, and physical mobility. Responses were on a 4-point Likert scale, with responses being: don‘t know, no improvement, some improvement, and a lot of improvement. Additionally, there were questions relating to whether staff perceived that clients disliked any technology items; barriers to clients using the technology; staff training; staff confidence when supporting clients; and barriers to staff supporting clients with the technology items.

### Interviews

Semistructured, once-off, one-on-one interviews were conducted by 1 of 2 University of South Australia RAs, with consenting staff. Interview questions were initially drafted by members of the evaluation project team and refined and co-designed in collaboration with the Stakeholder Advisory Group (Multimedia Appendix 1). Interviews were conducted either in person at the Social Day Program or over the phone. All interviews were recorded and then transcribed verbatim.

### Data Analysis

Observation and survey data are reported as counts or counts and percentage responses as relevant. Content analysis of open-ended survey questions was intended; however, an insufficient number of responses was provided, and a descriptive approach to reporting the responses was instead used. Reflexive Thematic Analysis [12,13] was used to analyze interview data, which were coded independently by a member of the evaluation team. Semantic codes and candidate themes were reviewed and discussed among the team across multiple iterations before the final themes were decided. We applied a constructionist epistemology, with an experiential lens on the data, with the intention of highlighting the experiences and perspectives of staff related to the use of technology in the Social Day Program environment. While our approach was predominantly inductive, in that we sought to represent the meaning of the information as it was communicated by the staff, elements of a deductive approach were applied to ensure that the established themes aligned with the research questions, for example, what the staff perceived to be barriers to and enablers of technology use in this setting.

### Ethical Considerations

This evaluation research was approved by University of South Australia’s Human Research Ethics Committee (204457). A waiver of consent was approved for the purpose of observing technology use in the Social Day Program. This was on the proviso that before commencing each observation period, staff and the evaluation project team announced to clients during Circle Time—an activity during which information is relayed to clients and their family members—that an observation period was about to begin. This enabled clients to leave the room if they did not wish to participate in this part of the evaluation project. Participants remained anonymous, in that information that might identify them was not recorded per their use of technology, and they were not asked to modify their behavior for the evaluation. Participants provided written informed consent for use of their survey data. Written informed consent was also provided by interview participants. Each interview participant received an honorarium (AUS $30 gift card; US $19.43) to acknowledge their contribution to the project. Data are anonymized and no information is provided that would identify participants.

## Results

### Participants

Thirty-one staff were approached to participate in the evaluation project; 19 based in the Care Hotel, and 12 staff based in the Social Day Program. Nineteen (61.3%) staff completed surveys (9 Care Hotel and 10 Social Day Program). Ten (32.3%) of the 31 staff approached (3 Care Hotel and 7 Social Day Program) provided written informed consent to participate in the interview; all 10 interviews were completed.

### Observation of Technology Use

Forty-eight observation periods were completed, totaling 126.5 observation hours. Technology use by clients was observed on 24 occasions, for 22 (17.4% of the observation time) hours. The relaxation chair and the robotic cat were the most frequently used technology items. The relaxation chair was used 7 times for a total of 9 hours and 36 minutes, and the robotic cat was used 7 times for a total of 3 hours and 14 minutes. Conversely, the Obie interactive games table and VR headsets were the least frequently used technology items (once for 64 min and once for 39 min, respectively). When technology was used, clients were mostly observed socially engaging and communicating with staff and other clients, with some clients also observed speaking to the robotic cat and dolls.

### Surveys

Of the 19 staff members who consented and responded to the staff survey, 10 (53%) worked in the Social Day Program and 9 (47%) were Care Hotel staff. Ninety-five percent (n=18) of staff reported that they helped or supported clients in using the technology items. Social Day Program staff assisted clients more than Care Hotel staff across all technology items except for the relaxation chair. Furthermore, all 9 Social Day Program staff indicated they assisted clients with the Obie interactive games table and robotic cat. Seventeen of the nineteen (89%) staff members nominated the VR headsets as the technology item they engaged with the least. Staff reported that on average, clients engaged with the technology items from 10-minutes to more than 60-minutes. This finding is reflected in the observation data, with the average time clients spent using technology items ranging from 32-minutes to 96-minutes.

Improvements in social engagement, cognitive awareness, communication, mood state, activities of daily living, and physical mobility were seen to some extent across each of the technologies, although not for all items (Table 1).

**Table 1.** Perceived improvements associated with technology use.

Technology item and domain		Don’t know, n (%)	No improvement, n (%)	Some or a lot of improvement, n (%)
Obie ICG^a^ table				
	Social engagement	1 (5)	3 (16)	15 (79)
	Cognitive awareness	3 (16)	4 (21)	12 (64)
	Communication	1 (5)	2 (11)	16 (84)
	Mood state	1 (5)	0 (0)	18 (95)
	Activities of daily living	2 (11)	9 (47)	8 (42)
	Physical mobility	0 (0)	8 (42)	11 (58)
VR^b^ headsets				
	Social engagement	10 (53)	4 (21)	5 (26)
	Cognitive awareness	12 (63)	5 (26)	2 (11)
	Communication	12 (63)	4 (21)	3 (16)
	Mood state	10 (53)	4 (21)	5 (26)
	Activities of daily living	11 (58)	7 (37)	1 (5)
	Physical mobility	12 (63)	6 (32)	1 (5)
Robotic cats				
	Social engagement	2 (11)	1 (5)	16 (84)
	Cognitive awareness	2 (11)	5 (26)	12 (63)
	Communication	2 (11)	2 (11)	15 (79)
	Mood state	2 (11)	0 (0)	17 (90)
	Activities of daily living	3 (16)	11 (58)	5 (26)
	Physical mobility	3 (16)	8 (42)	8 (42)
Robotic dolls				
	Social engagement	1 (5)	1 (5)	17 (90)
	Cognitive awareness	1 (5)	4 (21)	14 (74)
	Communication	3 (16)	1 (5)	15 (79)
	Mood state	2 (11)	0 (0)	17 (89)
	Activities of daily living	5 (26)	8 (42)	6 (32)
	Physical mobility	4 (21)	6 (32)	9 (47)
Relaxation chair				
	Social engagement	2 (11)	7 (37)	10 (52)
	Cognitive awareness	4 (21)	5 (26)	10 (53)
	Communication	3 (16)	5 (26)	11 (58)
	Mood state	1 (5)	0 (0)	18 (95)
	Activities of daily living	3 (16)	6 (32)	10 (53)
	Physical mobility	2 (11)	6 (32)	11 (58)

a ICG: interactive computer gaming.

b VR: virtual reality.

Nearly three-quarters (n=14) of the responding staff perceived there to be barriers to the clients’ use of technology, with equal numbers responding “yes” when compared by staff type. Ten (53%) of the responding staff had received training. Ten (53%) staff indicated they felt confident when assisting clients to use the technology; however, ten (53%) staff also felt that barriers existed that inhibited them from assisting the clients to use the technology (5 each from the Social Day Program and Care Hotel).

Key concepts raised by staff in their responses to the open-ended questions about their perceptions of technology use within the Social Day Program related to why clients may dislike technology, the barriers to technology use by clients, factors related to staff confidence in supporting clients to use technology, and the barriers to staff supporting clients to use technology.

Staff perceived that clients may dislike the technology because they have health or cognition issues that limit their ability to engage with technology; due to negative perceptions or stigmatization by other clients of people who use the robotic doll or cat; that clients have no interest in technology or use of technology; and that clients did not like particular items, finding them to be noisy or creepy (ie, the robotic doll). These aspects were also considered to be barriers to clients’ use of technology, with the addition of staff knowledge and confidence in the use of technology, and the time impact on staff to assist clients in using the technology.

### Interviews

Interviews were conducted with 12 staff members to assess the use of technology within the Social Day Program, and the enablers of and barriers to technology use. Interviews ranged between 18 and 44 minutes. The three themes developed during the analysis of the interview data: (1) technology has potential but is not for everyone, with the subtheme technology as a placation tool; (2) staff knowledge and confidence in using technology; and (3) technology functionality and support, are based on our interpretation of how staff experienced technology use in the Social Day Program and the factors that are likely to be enablers or barriers that influence the adoption of technology and sustainability of technology use in this environment. These themes address the research questions that form the basis for the evaluation. Quotes from participants are used as illustrative examples of the themes established.

### Technology Has Potential but it Is not for Everyone

This theme reflects the value and appropriateness that the staff perceive in technology use for their clients, assuming who might be likely to benefit from technology use and, conversely, for whom technology is not for. This theme incorporates the safety and personal considerations that were voiced as being needed when using technology. There were conflicting perspectives related to the use of technology within the Social Day Program. The potential benefits of the technology were raised often, including increased social engagement through the use of interactive games or being able to placate agitated clients with the robotic cat or time in the relaxation chair (a subtheme). While there was some concern about the cognitive benefits of the technologies, that the clients had to sit for prolonged periods to use some of them and therefore were being sedentary just as they would be at home (staff 14), and that some items had the potential to cause “sensory overload” (staff 2), others thought that the technology promoted social interaction, could “enrich the time clients spent at the Social Day Program” (staff 24), and could benefit future clients, in particular those who were tech-savvy. For one staff member, their initial perception that technology would “not be a great idea” (staff 1) changed positively when they saw the impact of technology use for some clients, for example, the connections made with the robotic cat. Similar observations of such connections are reflected by the following comment from a staff member.

I wasn’t sure about the babies and the cat. I thought maybe that was a bit babyish...It’s funny to watch how they [clients] perceive that and how quick they are to defend the cat, whereas sometimes you wouldn’t even get a couple of words out of them, but they’ve got to make sure the cat is okay.

[Staff 7]

Generational factors, in that the client demographic needed assistance as they were not tech-savvy, or not familiar with, or not interested in technology, situated some clients in the “technology is not for everyone” group, in the eyes of the staff. These factors were often raised as a possible barrier to greater use of the technology in the Social Day Program. Future generations were viewed to be more likely to be receptive to technology, as evidenced by the following comments:

I think it’s a double-edged sword and it has the potential to be brilliant, however unfortunately with that particular generation, because they were not particularly technologically minded or advanced, they are somewhat overwhelmed by it perhaps.

[Staff 6]

Look, we do have to help them most times just because they are technologies and it’s not their era. Today’s kids will be great in the future for these things, but these people were not brought up with these technologies.

[Staff 4]

While there was acknowledgment of the potential benefits of technology use, it was clear that staff were wary of the possible downfalls associated with it, demonstrating concern around physical and psychological safety for clients with frailty or cognition issues, or for whom memories of past traumas were triggered by using technology. Examples of this included clients who had worked in child abuse situations and had experienced miscarriages or the death of infants being exposed to the robotic dolls.

Some clients are really not suitable for the dolls and some aren’t suitable for the animals. It does depend, because they’ve got either histories of – we’ve got some clients that have worked in child abuse situations. They’re not good with the dolls, because it really upsets them. Knowing that background as well...we do the assessments and we ask the questions. But it’s good knowing those things.

[Staff 4]

The juxtaposition between the potential benefit of technology use and its appropriateness was evident in the commentary around the use of the VR equipment. There was a common thread that the VR equipment could be the most beneficial for clients of all of the technologies introduced to the Social Day Program, allowing clients to reminisce about places they may have previously lived in or visited and activities previously performed, such as flying planes. For others, the VR was a source of concern; for example, clients with frailty issues may be scared to use it due to a fear of falling, and those with cognitive issues may be confused by what they are seeing.

I think that the VR would definitely be useful for some clients who may enjoy it, a lot of more cognitive ones I think, because people who have low cognition may get very frightened of some of the things they see.

[Staff 1]

Ensuring the safety of clients while they were using technology by having measures in place to assess physical capacity to use the VR, for example, or having background information that enabled identification of potential trauma triggers to avoid clients being exposed to them, was raised, with some staff indicating processes were in place within the organization to accommodate this.

We’ve got a safety test before we just chuck anybody on it [VR], because some people do have the same thing as me with the nausea and dizziness and stuff. There is a possibility of falling down.

[Staff 4]

Combined, not being tech-savvy, being averse to technology, or perceived to be at risk of physical or psychological injury due to technology use cemented the concept that technology is not for everyone involved in the Social Day Program.

### Technology as a Placation Tool

Incorporated within this theme is the subtheme of technology as a placation tool. We initially wrestled with whether the use of technology in this way was situated as a potential benefit of technology use or a standalone theme; however, throughout the data, the utility of technology as a method of placation for clients, and therefore, it being perceived as a potential benefit, was evident. It seemed that while this was not an intended application of technology within the Social Day Program, placating agitated clients with items such as the robotic cat or time in the relaxation chair was rationalized by staff to lead to enriched experiences for those clients, as well as other clients in the program, whose experience was no longer being impinged upon by an agitated client. As a by-product, the benefit was extended to family members of the agitated clients who did not have to pick clients up from the Social Day Program earlier than intended, as described in the following comment:

It makes them extend their stay here in the day program. Rather than having to be picked up at lunchtime because their day is done and they’ve had enough stimulation for the day, they can be in there [the sensory room] for half an hour to an hour, come back out and enjoy the rest of the day ‘til 3:30 [PM].

[Staff 4]

### Knowledge and Confidence in Using Technology

This theme encapsulates concepts related to staff knowledge of the capabilities of the various pieces of technology and how to use them, as well as their confidence in assisting clients to use the technology, as a key driver in the use of technology in the Social Day Program. Further, these aspects are likely to influence whether technology continues to be used in the program. It was apparent across the data that training was important to staff; however, most reported that they had received little to no training in the use and maintenance of the technology. Further, there were perceptions that some of the technology required little training, such as the robotic doll and cat, whereas other types of technology, such as the VR or Obie, required more training of staff. This may have contributed to lower usage of these technologies as evidenced through observation, survey, and interview data, as may have confidence in using the technology. Perceptions of the relationship between training and confidence to use technology are contrasted. In some cases, staff reported that more training in the use of the available technology would increase their confidence to use the technology, for example:

I would like more training and capacity. I can do basic things, but I’m not confident using things...Yeah, I think I don’t feel really confident with it. I’d like to be. I’d like to learn to do it.

[Staff 9]

Others felt confident to use the technology, irrespective of the level of training they had received. It was suggested that increasing confidence in the use of technology may increase the enthusiasm staff have for technology use, and subsequently, increase the regularity of technology use in the programs at the organization and assist in better tailoring technology use to individual clients.

### Technology Functionality and Support

This theme reflects the importance of having available technology that is functional, as well as accessible support for staff in cases when the technology was not working. This would seem obvious in any environment where technology such as that implemented was being used; however, it was frequently raised by interviewees that technical and maintenance issues with the technology were common. These are clear barriers to the use of technology in the Social Day Program and are likely to influence the willingness of staff to use the technology or assist clients to use it. An example of this related to the VR technology, which staff reported had rarely been in working condition since its introduction. While some staff reported helping other staff to use the technology, obtaining higher-level support to repair the equipment had proved problematic, as illustrated by this comment from a staff member.

[If] I had a problem with something over the weekend let’s say, I would have absolutely no idea who to call to get that support and I don’t even think that the support necessarily is available. Therefore, from that perspective those clients would simply miss out until someone was able to be contacted to fix it. It would be a too bad, so sad kind of concept, which is not cool really.

[Staff 6]

## Discussion

### Principal Findings

This project aimed to undertake a multifaceted evaluation of the implementation of technologies at a Social Day Program to help inform decision-making regarding their implementation at the organization’s other sites. The evaluation included (1) observation of use, (2) surveys of staff members’ perceptions of enablers and barriers to use of the technologies, and (3) interviews with staff to further explore their experiences in use of technology and enablers and barriers to implementation and use of the technologies. The observation, staff survey, and interviews provide a rich dataset to enable the evaluation. Observation over an extensive period identified limited use of technology, further verified by the survey data, with potential reasons for this, including barriers to and enablers of technology use identified in survey data and thematic analysis of interview data.

When technology was used it was predominantly used during the Social Day Program and by Social Day Program clients rather than after hours by the Care Hotel guests. One reason for this may be that guests in the Care Hotel might be recuperating from medical procedures, rather than solely attending for respite purposes. As such, these guests may not be able to engage, nor be interested in engaging, in technology use at this time. Alternatively, the technology located in the guests’ Care Hotel rooms, such as an interactive television and tablet, which were not the focus of this evaluation, may have negated the need to access the technology located in the Social Day Program. For example, guests could relax and listen to music in their hotel rooms, and therefore would not need to access the sensory room and relaxation chair.

While items such as the robotic cat have been shown to be popular, a large portion of the use appears to be as a means of placating clients who were agitated or were disturbing other clients. This is not to say that the potential benefits of technology use at the organization were not recognized by some staff; however, encouragement of use may not be aligned with the original intention behind the implementation of the technology. Staff highlighted occasions when the use of the robotic cat by clients enhanced interaction between those clients, other clients, and staff. While mixed findings have been reported concerning the use of robotics in other aged care environments, some studies have shown that robotic animals such as cats and dogs can be conversation starters and contribute to enhanced engagement for older adults and people living with dementia [14,15].

In other cases, staff spoke of the opportunity that the sensory room provided for clients in being able to support an extended period of attendance at the Social Day Program, and in their eyes, held this as a potential benefit of technology use. Clients who may normally need to be picked up early, as they found the activities of the Social Day Program tiring or overwhelming, could instead remove themselves from the activities and take some time-out to relax in the sensory room. Being able to do this meant that these clients could spend longer days at the Social Day Program, rather than having to go home early. This has flow on benefits for their family carers, who, as a result, have a longer period on that day for respite. Research supports a reduction in care-related stressors for the carer during respite, and that the longer the respite duration, the greater the benefit to the carer [16]. Each of these factors can be highlighted to staff as potential benefits as a means of supporting implementation and greater use of the technology.

Harnessing the staff members’ own perceptions of the emotional, social, cognitive, and physical benefits of technology use that were identified through this evaluation may be one approach to facilitating successful implementation. Observational, survey, and interview data indicated that technologies were perceived to positively impact the emotional state of clients. This was not only the calming effect reported and that is acknowledged in the literature [17], but also included observed happiness and enjoyment, as well as the social engagement that occurred among groups of Social Day Program clients as they used the Obie and robotic cats, for example.

Safety—both physical and psychological—was a prominent consideration for staff. Caution was raised about the risk of falls with some of the technology, in particular VR. The use of VR has been evaluated in other aged care environments and has been shown to have both positive and negative impacts. While it has the potential to engage and enrich the experience of the organization’s clients by providing them access to activities, experiences, and environments that may no longer be physically accessible to them, VR may not be acceptable to, nor appropriate for, all people living with dementia [18]. Raised in the survey and interview data, these safety concerns extended to the possibility of psychological trauma for clients exposed to technology such as the robotic cats, categorizing them as part of the “technology is not for everyone” group. Concerns were raised about the stigma associated with people who use technologies such as the robotic cat or doll, because they are “not real.” “Infantilization” of older adults living with dementia and their loss of dignity through the use of robotic toys, which they perceive to be real, has been explored in other research [17], and in some cases, it is the family members of the older adult holding this perception [19]. Strategies suggested to minimize this and maintain dignity when items such as robotic toys are used to engage people include creating an obvious environment of “play” [20].

### Barriers to, and Enablers of, Technology Uptake and Use

As evidenced through this evaluation, several factors act as barriers to and enablers of technology use within the Social Day Program. The key factors, reflected by the developed themes, relate to staff training in, and knowledge of, technology operation and features; technology functionality and the availability of real-time technology support for staff; and perceptions of a generational influence on technology use. All of the barriers—and subsequently the enablers—to technology use identified in this evaluation were common to another recent Australian evaluation of technology use in aged care [9], suggesting they are not specific to this particular organization, but more reflective of the aged care environment generally.

Staff awareness and understanding of the underlying purpose of technology use for this population, beyond it being used as a behavior management tool, may also act as an enabler of greater use of technology within the organization. This includes opportunities for technology to promote “meaningful engagement” and social interaction [19], particularly, the technology should facilitate social interaction among those present and not be a replacement for it [9]. However, technologies that require staff to invest more of their already limited time to support clients in using them may also be less likely to be encouraged within the Social Day Program.

“Visibility” of the technology was considered by some staff to be an enabler of technology use; however, it was not raised sufficiently to warrant inclusion as a theme. Staff suggested that technology items that are visible and easily accessible by staff and clients are more likely to be used regularly. Further, staff members who regularly use the technology and encourage clients to do so have the potential to encourage other staff members to do the same. As such, increased visibility of the technology has the potential to encourage more use within the organization.

Other potential barriers to the use of technology relate to perceptions that the current generation of clients at the Social Day Program are not “tech-savvy,” do not like technology, or just have no interest in using it. Interviews identified that the clients were perceived not to have used much technology across their lifetime and that technology is likely to be more acceptable to future generations of clients who have grown up with and are familiar with technology. Staff reported observing positive impact related to social engagement, communication, and mood state, suggesting that some clients did enjoy using the technology. Enjoyment and acceptability of technology by older adults would seem to be obvious in whether technology is used and have been shown to be perceived by older adults as relevant in the successful implementation of technology to support ageing in place [20].

### Sustainability

Targeting the enablers of technology use identified in this evaluation will contribute to ongoing technology use in the organization’s Social Day Program. Taking a person-centered approach to technology use, by modifying organizational approaches to understanding the interests of clients, the physical and cognitive capabilities of clients, knowledge of potential triggers for negative or trauma responses associated with technology use; as well as technology that is maintained in good working order will also be a driver of sustainability.

### Limitations

A limitation of this evaluation was having little data from clients and their family members, and therefore not being able to make their voices prominent in this evaluation. To an extent, this is reflective of other evaluations that have been undertaken in the aged care environment. In some cases, family members may not be involved in the lives of their guest or client family member. In others, the only respite family members may have is when the guest or client is at the Social Day Program. This may see family members not wanting to use that time for activities such as this evaluation.

In cases where guests or clients are living with cognitive decline or dementia, it was difficult to engage them in the interview process. However, it is important to ensure that people living with cognitive decline or dementia are included in projects about them, in particular, in the consultation and co-design process [21], which we were able to achieve through having clients and family members in the stakeholder group. While there were several instances in which staff provided negative feedback related to the technology not being operational, with limited support provided, it is also possible that other staff were reluctant to provide negative perspectives, which may introduce some bias to the findings.

### Recommendations

While the use of technology may benefit this population, the potential benefits have not been fully realized at the organization due to several implementation barriers: there is a lack of staff knowledge, confidence in use, and training relevant to the operation of the different technologies, the technology has not always been operational and ongoing, accessible support is not available. There is a perception that technology has benefits, although the use of technology may not always be as intended by the organization, however, this is contrasted by the expressed view that technology is not for everyone and there are physical and psychological risks involved in use of technology in the Social Day Program population, many of whom live with cognitive decline. By addressing the barriers identified in this evaluation and strengthening the enablers per the following recommendations, the potential for clients to benefit across the domains could be enhanced.

Based on the results from the evaluation and considering the evidence from the literature, the following recommendations may enhance the implementation of technology in programs similar to the one described here:

- Increase staff knowledge of technology and its potential benefits for older adults living with cognitive decline, through the provision of more structured and regular training, a summary information sheet of project findings, quick refresher sessions, instruction sheets, and time to “play” and practice with the technology. This needs to include not only how to operate the technology and trouble shoot common problems or issues, but also provide an understanding of all the features and options (to enable tailoring to client needs or capabilities) and how to use the technology to best benefit the clients.
- Develop an information technology maintenance and support process that is resourced to ensure the equipment is functional at all times and enables staff to access real-time information technology support to address operational or technical issues. This should also include the development of comprehensive but user-friendly instructions to assist with understanding the features of the technology, operating the technology, and troubleshooting.
- Ensure that appropriate measures are in place to establish client physical and psychological safety, so that they are not exposed to technology that may be inappropriate for their circumstances.
- Increase the visibility and accessibility of the technology, so that it is at the forefront of staff’s, clients’ and their family members’ or carers’ thoughts as an activity. This may be through pictural posters placed around the room as reminders that the technology is there, with simple step by step instructions placed near technology or by leaving the technology in easy-to-access locations.
- Consider the placement of technology within the space.

### Conclusions

This evaluation of technology use in a Social Day Program has identified that technology is not being used extensively, nor is it being used for the purposes of enrichment or experience enhancement. Multiple barriers to implementation and sustained use of the technology items have been identified, spanning perceptions of clients’ preference or low ability, to a lack of staff training and knowledge to support adequate use of the technology. Recommendations to improve implementation and promote sustained use of technology, based on the findings of this evaluation and evidence from the literature, may apply to other organizations seeking to implement these technologies in similar programs.

## Supplementary material

10.2196/60297 Multimedia Appendix 1 Semistructured interview guiding questions: family member or carer.

## Abbreviations

RA: research assistant
VR: virtual reality
