# Supplementary material for: Implementation of New Technologies in an Aged Care Social Day Program: Mixed Methods Evaluation
Source: JMIR Aging. 2025 May 12;8:e60297. doi: 10.2196/60297 (PMC12088610; doi:10.2196/60297)
Supplement: Multimedia Appendix 1 [file aging-v8-e60297-s001.docx]

**Background:**

How long has your family member/the person you care for been attending the Care Hotel or Social Day Program?

How regularly and in what capacity do they attend?

What is their functional and cognitive status?

**New Technology:**

What are your perceptions of new technology?

Do/did you think that the new technology could have an impact on your family member/the person you care for?

Which technologies has your family member/ the person you care for, been using?

Do you think the ….(specify the technology indicated) has had any effect on ... social engagement

Do you think the ….(specify the technology indicated) has had any effect on … communication,

Do you think the ….(specify the technology indicated) has had any effect on … cognitive awareness,

Do you think the ….(specify the technology indicated) has had any effect on ... activities of daily living

Do you think the ….(specify the technology indicated) has had any effect on ... mood state

Do you think the ….(specify the technology indicated) has had any effect on ... behaviour.

Do you think the new technology has been of benefit to your family member/person you care for?

Do you think there are any barriers to the use of the new technologies?

Which ones? And in what way?

***End of interview question guide for Family Member/Carer.***

**Semi-structured interview guiding questions: Staff**

**Background:**

What is your role at the Care Hotel/Social Day Program?

How long have you been in your current role at the Care Hotel/Social Day Program?

**New Technology:**

What are your perceptions of the new technology? Did you think that the new technology could have an impact on the guests/clients? If so, what were your initial thoughts? If not, why not?

Do your day-to-day work tasks/responsibilities involve you supporting the guests/clients to use the technology? Please can you explain how?

Do you consider supporting the guests/clients to use the technology as being a part of your role? Do you feel it should/shouldn’t be a part of your role?

What technology did you receive training on?

And, what was the training? (i.e., webinar, hands on, manual, learn by use)

What guests’/clients’ likes/preferences are taken into consideration in the use and setup of the technology? Please can you explain how? (i.e., to ascertain individual’s background, history and if this is taken into consideration when setting certain features of technology)

What level of free choice are the guests/clients provided in choosing particular games/settings/features? Are games/technology etc. pre-selected or allocated by staff at the beginning of the day?

What barriers or challenges do you face in helping the guests/clients to use the technology?

What time(s) of the day have you observed the guests/clients using the technology most frequently?

Which technologies have you observed the guests/clients using?

Which technologies have you observed being used by guests/clients attending the Social Day Program? Have you observed guests/clients using any other technology outside of those offered in the Social Day Program (e.g., in the Care Hotel)?

Which technology do you think is used the most?

Which technology do you think is the most beneficial to the guests/clients?

What effect does ….(specify the technology indicated) have on the guests’/clients’ social engagement?

What effect does ….(specify the technology indicated) have on the guests’/clients’ communication?

What effect does ….(specify the technology indicated) have on the guests’/clients’ cognitive awareness?

What effect does ….(specify the technology indicated) have on the guests’/clients’ Activities of daily living?

What effect does ….(specify the technology indicated) have on the guests’/clients’ mood state?

What effect does ….(specify the technology indicated) have on guests’/clients’ behaviour?

What barriers and/or challenges do the guests/clients face in using the new technology?

What technology do the guests/clients face biggest challenges?

***End of interview question guide for Staff.***
